# Ultrasound deep learning features for predicting cervical lymph node metastasis in papillary thyroid carcinoma

**DOI:** 10.3389/fonc.2026.1721234

**Published:** 2026-05-20

**Authors:** Xinxin Li, Yin Zhu, Ying Wu, Qi Zhou, Zakari Shaibu, Liang Yin, Qing Cang

**Affiliations:** 1 Department of Otorhinolaryngology – Head and Neck Surgery, Affiliated People’s Hospital of Jiangsu University, Zhenjiang, China; 2 Department of Oncology, Jurong Hospital Affiliated to Jiangsu University, Jurong, Zhenjiang, China; 3 Department of Breast Surgery, Affiliated People’s Hospital of Jiangsu University, Zhenjiang, China; 4 School of Medicine, Jiangsu University, Zhenjiang, China; 5 Department of Medical Oncology, Affiliated People’s Hospital of Jiangsu University, Zhenjiang, China; 6 Department of Ultrasound, Jurong Hospital Affiliated to Jiangsu University, Jurong, Zhenjiang, China

**Keywords:** cervical lymph node metastasis, deep learning radiomics, papillary thyroid carcinoma, recursive feature elimination, thyroid cancer

## Abstract

**Background:**

The purpose of this study was to create and validate a clinical deep learning radiomics (DLR) model for determining cervical lymph node metastasis (CLNM) status in patients with papillary thyroid cancer (PTC).

**Methods:**

A total of 205 eligible patients with PTC who underwent preoperative thyroid ultrasonography (US) between January 2015 and April 2020 were retrospectively enrolled. The training cohort consisted of 143 patients, while the validation cohort included 62 patients. A DLR model was built using deep learning features, a clinical model was built using clinical parameters, and a Cli-DLR model was built using both DLR features and clinical factors.

**Results:**

In the validation cohort, the Cli-DLR model performed well, with an AUC of 0.80. The Cli-DLR model performed well in terms of precision-recall and calibration curve analysis. Furthermore, the Cli-DLR model outperformed experienced radiologists. This approach has the potential to help guide optimal CLNM management in PTC patients, particularly by preventing overtreatment.

**Conclusions:**

In conclusion, we developed a Cli-DLR model to assess the cervical lymph node status of PTC patients prior to surgery. The Cli-DLR model outperformed radiologists and the clinical model. Consequently, this model can provide a possible noninvasive method for detecting CLNM and aid in clinical decision-making due to its favorable specificity and sensitivity. High-level evidence for clinical use in later studies is anticipated to be obtained through prospective multicenter validation.

## Background

Recently, there has been a notable increase in the number of thyroid cancer cases globally, with papillary thyroid carcinoma (PTC) being the most common type (1, 2). PTC constitutes more than 80% of thyroid cancer cases, with a portion of these cases progressing to cervical lymph node metastases (CLNM) (3–6). Pathological analysis revealed that lymph node metastasis (LNM) occurs in approximately 30% to 80% of patients with PTC (7, 8). It is viewed as a factor that increases the risk of local recurrence and distant spread of cancer and lowers survival rates (8–10).

Considering the significant clinical risk linked with positive lymph nodes in patients with PTC, some researchers have suggested performing routine cervical lymph node dissection (CLND) during the initial surgery as a means to enhance treatment results (11). During PTC treatment, the presence of CLNM could influence the surgical method and postoperative assessment. Employing a consistent surgical approach and strategy for CLND might increase the success rate of treatment and lower complication rates. Thus, precise preoperative evaluation of CLNM is vital because it enables clinicians to devise surgical strategies and predict patient prognosis effectively.

Ultrasound (US) is the prevailing approach for assessing lymph nodes before surgery in patients with PTC (12). However, its sensitivity in identifying CLNM ranges from only 26–47%, which is insufficient for accurate assessment (13).

US has limitations in regard to imaging deep structures or those obstructed by air or bone, such as in cases of morbid obesity, restricted neck movement, or distant cervical adenopathy.

Therefore, it is essential to conduct a more precise evaluation before surgery to detect CLNM in patients with PTC to minimize the need for unnecessary CLND. Radiomics offers the ability to automatically extract numerous quantitative image characteristics from medical images, which may be difficult for the human eye to identify (14, 15). The drawbacks of manually crafted features are rooted in the need for human labelling and their inability to adapt precisely to a particular task (16). In contrast, deep learning radiomics (DLR) (15) represents an inventive approach capable of autonomously uncovering various levels of representations tailored to specific prediction tasks through end-to-end learning. In contrast to radiologists who consider both clinical data and US information to reach diagnoses, most artificial intelligence (AI) models typically yield results without disclosing their reasoning process.

The absence of clear information is seen as a factor contributing to radiologists’ doubts regarding the practical use of AI models in clinical settings (1). DLR combined with clinical data such as age, sex, tumor size, and other network characteristics enhances the model by incorporating additional information alongside image features from US, thus boosting overall model performance through the collaborative utilization of clinical data and deep image features (17) and the approval of radiologists. Therefore, this study aimed to assess how effectively a combination of clinical and US features, along with the DLR, can predict the degree of CLNM involvement in PTC patients.

## Materials and methods

### Patients

**Figure 1:**
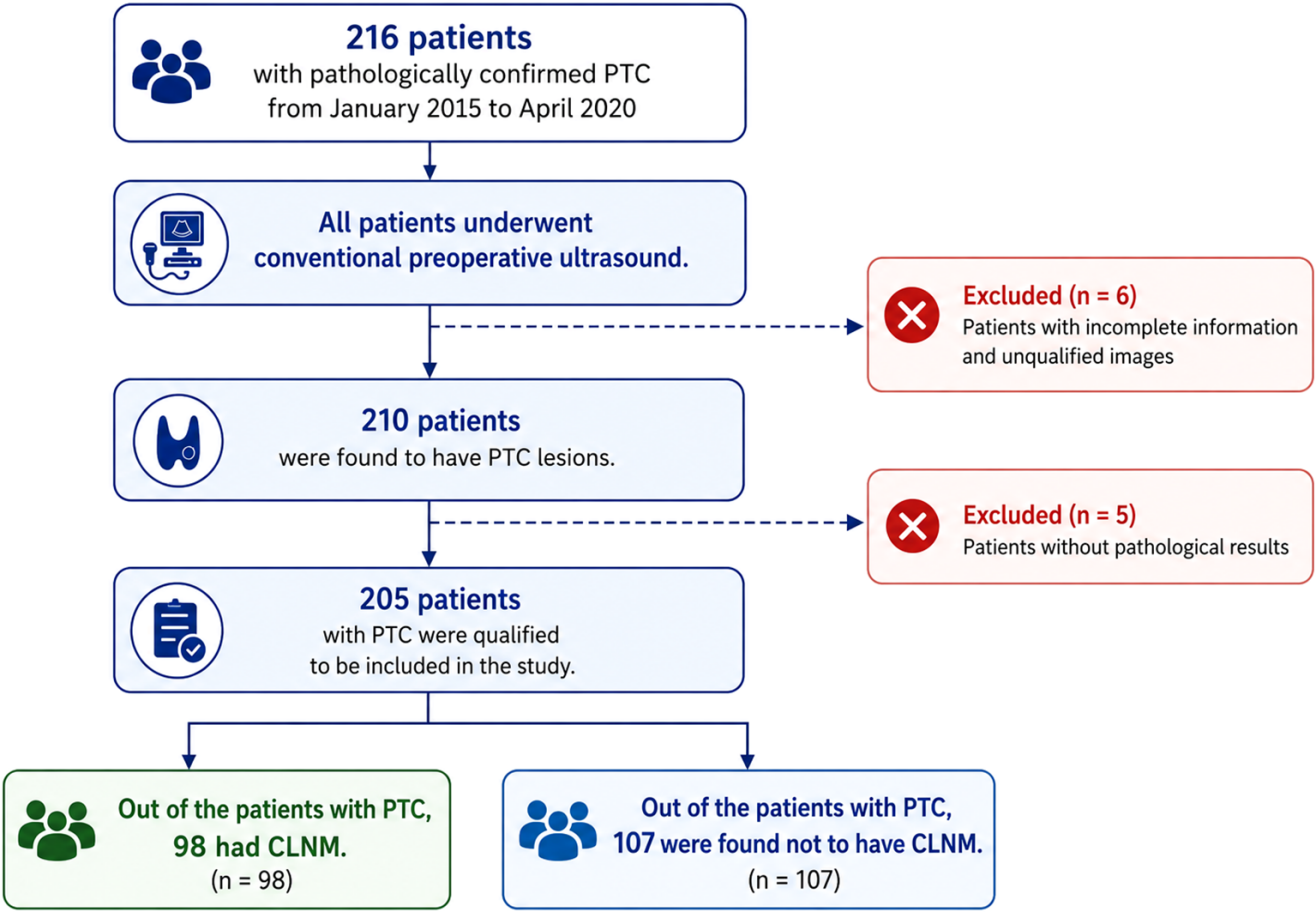
Schematic diagram of patient selection. PTC, papillary thyroid carcinoma; CLNM, cervical lymph node metastasis.

**Figure 2:**
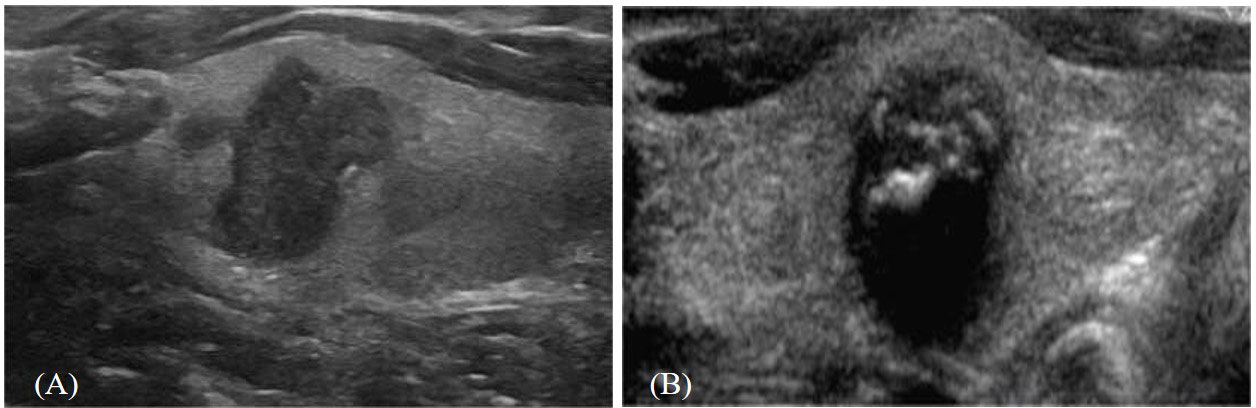
Ultrasound conventional B-mode images of papillary thyroid carcinoma. **(A)** A distinctly hypoechoic, heterogeneous solid nodule exhibiting irregular, ill-defined margins and a taller-than-wide configuration, devoid of discernible calcifications. **(B)** A markedly hypoechoic solid nodule with irregular margins, exhibiting internal echogenic foci consistent with microcalcifications and posterior acoustic shadowing, with a taller-than-wide shape.

This study was approved by the institutional review board of Jiangsu University Affiliated People’s Hospital and complied with the ethics standards of the Declaration of Helsinki. The requirement for informed consent was waived owing to the retrospective nature of this study. The inclusion and exclusion criteria are shown in **Table 1**. **Figure 1** illustrates the flowchart detailing the process of patient recruitment. Over the period from January 2015 to April 2020, a total of 205 PTC patients from our hospital were retrospectively selected. Each patient underwent routine 2‐dimensional US examination (**Figure 2**). Based on pathological findings, the patients were categorized into groups: those with CLNM and those without CLNM. The patients included in the study were then split randomly into a training cohort comprising 143 patients and a validation cohort comprising 62 patients at a ratio of 7:3.

**Table 1:** Inclusion and exclusion criteria.

Inclusion and exclusion criteria
Inclusion criteria
1. nodules that had clear surgery‐ and puncture biopsy pathology‐confirmed PTC
2. routine US, with complete images, clear quality
3. did not receive chemoradiotherapy or other cancer treatment prior to surgery
Exclusion criteria
1. pathology results that could not be identified as PTC nodules
2. unclear images with incomplete nodules
3. Pregnant and lactating women
4. patients with a severe allergic history or severe cardiopulmonary disease

US; ultrasound.

### Clinical parameters

The preoperative clinical parameters collected for analysis included clinical characteristics and US findings of the thyroid. Clinical characteristics included age and sex, and the US characteristics included thyroid tumor size or mass (maximum long axis of the nodule), tumor location (left lobe, right lobe, or isthmus), tumor position (upper, middle, or lower pole), aspect ratio (≤1 or >1), internal echo pattern (uniform or nonuniform), tumor border (clear, less clear), shape (regular or irregular), US ETE diagnosis (without ETE or with ETE), tumor peripheral blood flow (without or abundant), and tumor internal vascularization (without or abundant).

### Ultrasound examination

All patients underwent routine US examination prior to surgery, which was performed by well‐trained radiologists who specialize in thyroid imaging and have over 15 years of clinical experience using a Philips Q5, Philips iU22 (both Healthcare, Eindhoven, The Netherlands) or a GE LOGIQ s8, LOGIQ E20, LOGIQ E9 (GE Medical Systems, American General, Boston, MA, USA) US system with a 5-12 MHz linear array transducer.

The patient lay on their back, without a pillow, allowing their head to be slightly lowered and reclined. This positioning aimed to maximize neck exposure for a thorough US examination of the thyroid and cervical region. The examination involved scanning longitudinally and horizontally, with a focus on meticulously inspecting lymph nodes across all neck regions.

### Construction of a deep learning model for DLR feature extraction

#### Data preprocessing and data augmentation

The imaging repository was systematically reviewed to identify US images of patients with PTC (**Figure 2**), and the presence or absence of CLNM was matched with the corresponding imaging data. All US images were independently evaluated by an experienced radiologist (with more than 15 years of thyroid US examination) for subsequent analysis. To minimize potential bias in image selection and feature extraction, the reviewer was blinded to all clinicopathological information, including lymph node status and postoperative histopathological findings.

Prior to preprocessing, grayscale conversion was applied to all B-mode US images to eliminate unnecessary image channels. Masses were detected by bounding box labels, and a radiologist with eight years of experience in US confirmed the annotations. The rectangular regions of interest (ROIs) were extracted from the original US images. Cropped US images were resized, padded, and normalized to 224 × 224 pixels, keeping their original aspect ratios, to minimize the influence of irrelevant background information.

A pixel border around the lesion zone was added to capture some of the surrounding area near the lesion, which could provide significant information and prevent insufficient mass extraction. To assess feature reproducibility and stability, ICC analysis was performed on 42 randomly selected images that had been independently segmented by another radiologist with over 15 years of thyroid US imaging experience. Features with ICC ≥ 0.75 were kept for further analysis, while those with ICC< 0.75 were removed to ensure model robustness.

The training dataset was used to optimize the model’s parameters.

All preprocessing steps were conducted in Python (version 3.10.12) by using Keras preprocessing.

#### Deep learning radiomic feature extraction

**Figure 3:**
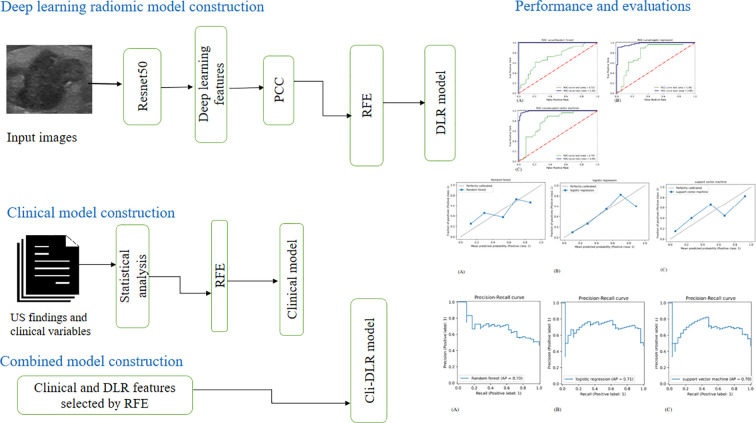
Flowchart of the model construction. Workflow of model construction for predicting cervical lymph node metastasis in patients with papillary thyroid carcinoma. RFE, recursive feature elimination; PCC, Pearson correlation coefficient; US, ultrasound; LASSO, least absolute shrinkage and selection operation; DLR, deep learning radiomics.

A flowchart of the study is shown in **Figure 3**. In our research, we utilized ResNet50 as the base model for extracting deep learning features. Previous studies have demonstrated the effectiveness of ResNet50 in yielding high-quality deep learning features (18, 19). Building upon AlexNet’s groundwork, ResNet was created and enhanced, showing benefits such as identity mapping and an impressive depth of 152 layers. Nevertheless, the challenge of vanishing or exploding gradients arises as the network depth increases (20–22).

To address this issue, methods such as normalized initialization and the incorporation of intermediate normalization layers have been introduced (21–24). Despite these efforts, as network layers stack, the accuracy of the training dataset may plateau or even decline, a phenomenon known as degradation. Notably, this decline is not caused by overfitting; rather, it is exacerbated by the addition of layers to the deep model (25, 26).

ResNet tackles this issue by employing a deep residual learning framework, effectively mitigating degradation (27). In this study, transfer learning was used to fine-tune all the weights and biases, considerably reducing the training time. The parameters were pretrained on the ImageNet dataset before being loaded and retrained on our dataset.

Finally, the original classifier for the ImageNet classes was replaced with a binary classifier, yielding a class probability vector ranging from 0 to 1 as the prediction result for each patient. The network was trained from scratch using the cross-entropy loss function and the Adam optimizer, with a learning rate of 0.0001 and a batch size of 32. The implementation of the training and validation codes utilized PyTorch version 2.2.2+cu118 and Keras version 2.10.0. The model underwent 200 epochs of training to prevent overfitting.

Given the limited training data available in this work and the necessity of reducing overfitting and sample imbalances, data augmentation was used. This strategy for augmenting datasets has proven useful. The generalizability of the model improves as the dataset size increases. When the dataset comprises a small number of samples, data augmentation improves the training data by modifying the current dataset (28).

Data augmentation was applied for the training cohort to reduce the potential bias caused by the limited number of images in the training procedure (29). US images in the training cohort were augmented through several random transformations, which meant ran -domly horizontal and vertical flipping the input image,randomly adjusting the height of an image by an amountof 0.2, randomly adjusting the width of an image by an amount of 0.2, randomly zooming into an image by anamount of 0.2, randomly rotating an image by an amountof 0.2. This process ensures that the model focuses on identifying thyroid lesions amidst potential noise sources.

In the ResNet50 model, the fully connected layer and softmax layer were removed, and the output values of the nodes in the last layer were used as the deep learning features. Ultimately, the ResNet50 model extracted 2048 deep learning features from each PTC US image.

### Feature selection and construction of DLR models

Because it is straightforward to implement and use, recursive feature elimination (RFE) is widely used in machine learning to choose the most important features from a training dataset for predicting the target variable (30). RFE boosts model effectiveness by prioritizing these relevant characteristics, reducing overfitting, and improving interpretability (30). The procedure involves gradually removing the least important elements until the target number of features is achieved, ultimately resulting in building a model with the remaining features (31).

To prevent bias and overfitting, the retrieved deep learning features were normalized with a MinMax scalar. Because the feature space is highly dimensional, the number of features must be minimized to avoid the interference of a large number of redundant features in the data analysis, which has an influence on model creation and increases computational costs.

We employed Pearson’s correlation coefficient (PCC) to minimize the row spatial dimension of the feature matrix, resulting in each characteristic being substantially independent of the training data. Any two features with a PCC greater than 0.85 were considered redundant. In the training cohort, CLNM-related predictive characteristics were selected via RFE with 15-fold cross-validation (**Supplementary Figure 1**).

We employed support vector machine (SVM), logistic regression (LR), and random forest (RF) classifiers to create prediction models based on RFE’s main features. During the validation step, the same feature sets were selected and fed into the model.

SVMs are capable of categorizing data points even when they are not linearly separable by employing a technique that maps the data to a high-dimensional feature space. This strategy, rooted in statistical learning theory and fundamental to SVM, utilizes a kernel function to project specific data points into this high-dimensional feature space, enabling the classification of data points that would otherwise be considered nonseparable in a linear context (32).

The linear SVM identifies the optimal hyperplane in the form described by Equation 1.

(1) $$f(x)=w^{T}x+b$$

where *ω* is a dimensional coefficient vector and b is an offset (33).

The LR technique involves estimating the probability of a discrete outcome based on an input variable. The most common application of LR is modelling binary outcomes, where the result can take two values, such as true/false or yes/no. In LR, the odds, or the likelihood of success divided by the probability of failure, are transformed using the logit formula. Equation 2 illustrates the logistic function (34).

(2) Logit(pi)=1/(1+exp(−pi))

RF is a supervised learning classifier that combines several decision trees on subsets of data to solve complex problems (35). By averaging the input from each tree to predict outcomes, the prediction accuracy improves and may be tailored to different learning requirements. By aggregating the outputs of all the regression trees, forecast the outcome on the unseen samples, x’, when the training phase is complete, as indicated in Equation 3 (36).

(3) $$f=\frac{1}{B}\sum_{b=1}^{B}f_{b}(x^{\prime })$$

### Feature selection and construction of clinical models

The study assessed basic clinical features and selected statistically significant features (P< 0.05). The RFE approach, with 15-fold cross-validation, was then utilized to select the most effective CLNM-related predictive features from the training dataset (**Supplementary Figure 1**). Prediction models based on essential RFE features were built using SVM, LR, and RF classifiers. In the validation process, the same feature sets were chosen and fed into the model.

### Construction of the combined model (Cli-DLR model)

To analyze the impact of DLR features on CLNM prediction, we built a separate model that was based on the clinical variables together with DLR features selected by the RFE using SVM, LR, and RF classifiers.

### Evaluation metrics employed in this study

The detailed description was presented in **Supplementary Method**. The models’ performance on both the training and validation datasets was evaluated using metrics such as the area under the curve (AUC), precision, sensitivity, specificity, negative predictive value (NPV), positive predictive value (PPV), accuracy, precision–recall curves, F1 score, and other standard clinical statistics. The best-performing model was selected from a pool of models based on its performance on the validation dataset, which was evaluated and compared for diagnostic efficacy.

### Statistical analysis

Statistical analysis was conducted utilizing Python (version 3.10.12) and IBM SPSS Statistics for Windows version 26.0 (Armonk, New York, USA). To compare differences in categorical characteristics, either Pearson’s chi-square test or Fisher’s exact test was used. For continuous variables with a normal distribution, the independent sample t test was utilized, while for variables without a normal distribution, the Mann–Whitney U test was applied. A two-sided P value<0.05 was considered indicative of a statistically significant difference.

The AUC, F1-score, recall, precision, sensitivity, specificity, accuracy, NPV, and PPV of each prediction model were computed using Scikit-learn version 1.2 (37).

## Results

### Clinical characteristics

A total of 205 PTC patients, ranging in age from 18 to 78 years, were enrolled; their average age was 47.22 ± 11.30 years, and their male to female ratio was 1:3.56. Of the patients, 107 had no CLNM, and 98 had CLNM confirmed. Using stratified sampling, every patient was randomized into two groups: a validation group (n = 62) and a training group (n = 143).

The clinical data for the CLNM and non-CLNM groups are shown in **Table 2**. There were notable differences between the two groups in terms of age, US CLNM diagnosis, mass, capsular invasion, shape, internal echo, aspect ratio, and tumor internal vascularization (all P< 0.05).

**Table 2:** Patient characteristics of the PTC patients in the CLNM and PTC without CLNM groups.

Characteristic	CLNM(-) (n=107)	CLNM(+) (n=98)	P
Age, mean ± SD, years	48.97 ± 10.81	45.31 ± 11.56	0.035^*^
Sex, n			
Female	80	80	
Male	27	18	0.154
Ultrasound Characteristic			
Tumor size(mass)	7.30 ± 5.01	11.57 ± 7.74	0.00^*^
Tumor location			
Left lobe	44	34	
Right lobe	40	37	
Isthmus	23	27	0.515
Tumor position			
Upper pole	53	45	
Middle pole	4	3	
Inferior pole	50	50	0.818
Internal echo pattern			
Uniform	26	13	0.033*
Nonuniform	81	85	
Tumor border			
Clear	30	29	0.463
unclear	77	69	
Tumor internal vascularization			0.001^*^
Without	71	42	
Abundant	36	56	
Tumor Peripheral blood flow			0.236
Without	61	50	
Abundant	46	48	
Ultrasound ETE diagnosis			0.009^*^
Without ETE	104	86	
With ETE	3	12	
Aspect ratio			0.000^*^
≤1	55	74	
>1	52	24	
Shape			0.025^*^
Regular	85	65	
Irregular	22	33	
Ultrasound CLNM diagnosis			0.035^*^
Without CLNM	68	49	
With CLNM	39	49	
Postoperative diagnosis			
capsular invasion			0.022^*^
Negative	63	43	
Positive	44	55	

ETE, extrathyroidal extension; CLNM, cervical lymph node metastasis; SD, standard deviation.

### Diagnostic performance of the clinical, DLR, and Cli-DLR models

**Supplementary Figure 1** shows the effects of applying RFE to clinical data and deep learning features. Nine clinical factors (**Supplementary Table 1**), thirty deep learning features (**Supplementary Table 2**), and thirty-nine features (**Supplementary Table 3**) were chosen for building the Clinical, DLR, and Cli-DLR models, respectively.

According to the clinical data, RF, LR, and SVM produced AUC values of 0.66, 0.63, and 0.57, respectively, in the validation cohort. Compared to the other classifiers, RF performed the best, with an AUC of 0.66. **Table 3** provides detailed information on the clinical models’ prediction performance. **Supplementary Figure 2** shows the ROC curves for the three classifiers used to generate the clinical models.

**Table 3:** Predictive performance of the machine learning models for the training and validation cohorts.

	Validation cohort							Training cohort						
	ACC	AUC	SEN	SPEC	PPV	NPV	F1	ACC	AUC	SEN	SPEC	PPV	NPV	F1
Clinical model														
RFE+RF	0.68	0.66	0.52	0.82	0.71	0.66	0.60	1.00	1.00	1.00	1.00	1.00	1.00	1.00
RFE+LR	0.60	0.63	0.52	0.67	0.58	0.61	0.51	0.72	0.79	0.70	0.74	0.71	0.72	0.71
RFE+SVM	0.56	0.57	0.41	0.70	0.55	0.58	0.47	0.69	0.77	0.65	0.72	0.68	0.69	0.67
DLR model														
RFE+RF	0.68	0.72	0.62	0.73	0.67	0.69	0.64	0.99	0.99	1.00	0.97	0.97	1.00	0.99
RFE+LR	0.73	0.76	0.76	0.70	0.69	0.77	72.00	0.96	0.99	0.96	0.96	0.96	0.96	0.96
RFE+SVM	0.76	0.78	0.72	0.79	0.75	0.76	0.74	0.95	0.99	0.93	0.97	0.97	0.94	0.95
Clinical-DLR model														
RFE+RF	0.68	0.72	0.66	0.70	0.66	0.70	0.66	1.00	1.00	1.00	1.00	1.00	1.00	1.00
RFE+LR	0.69	0.80	0.69	0.70	0.67	0.72	0.68	0.95	0.98	0.91	0.99	0.98	0.92	0.95
RFE+SVM	0.71	0.79	0.69	0.73	0.69	0.73	0.69	0.95	0.99	0.96	0.97	0.97	0.96	0.96

AUC, area under the curve; ACC, accuracy; SEN, sensitivity; SPEC, specificity; NPV, negative predictive value; PPV, positive predictive value; RFE, recursive feature elimination; DLR, deep learning radiomic; SVM, support vector machine; RF, random forest; LR, logistic regression.

With respect to the DLR model, RF, LR, and SVM on the DLR data had AUC values of 0.72, 0.76, and 0.78, respectively, in the validation cohort. Compared to the other classifiers, the SVM had the highest AUC of 0.78. **Table 3** provides detailed information on the DLR models’ prediction performances. **Supplementary Figure 3** shows the ROC curves of the three classifiers used to construct the DLR models.

**Figure 4:**
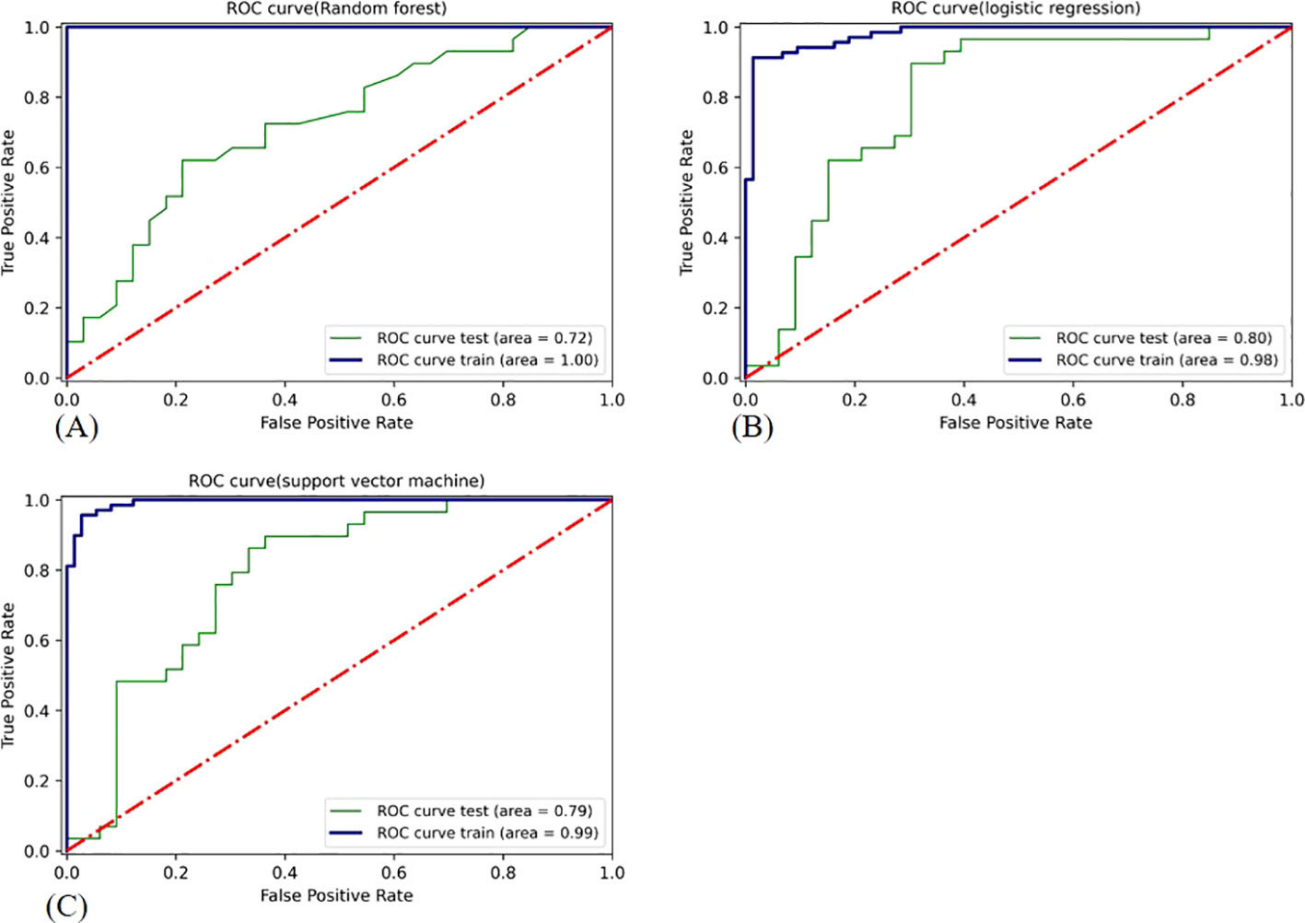
ROC curve of the classifiers used in building the Cli-DLR model employing RFE. ROC, receiver operating characteristic. **(A)** Random forest; **(B)** logistic regression classifier; **(C)** support vector machine.

CLNM prediction in our validation cohort was significantly enhanced when selected DLR features were added to the selected clinical parameters via RFE, as opposed to the clinical model without DLR features. In the combined model, RF, LR, and SVM had AUC values of 0.72, 0.80, and 0.79, respectively. Compared to the other classifiers (**Figure 4B**), the LR classifier performed the best, with an AUC value of 0.80. **Table 3** provides detailed information on the prediction performance of the Cli-DLR model. **Table 4** shows the DeLong test results for the models. **Figures 4A-C** shows the ROC curves for the three classifiers used to generate the Cli-DLR models.

**Table 4:** Comparison of the models using DeLong test.

Model 1	Model 2	AUC 1	AUC 2	AUC difference	Z-score	P-value	Significant
Cli_DLR_model	DLR_model	0.801463	0.776385	0.025078	0.471536	0.64	False
Cli_DLR_model	Clinical_model	0.801463	0.659352	0.142111	4.004371	0.00	True
DLR_model	Clinical_model	0.776385	0.659352	0.117032	15.22366	0.00	True

**Figure 5:**
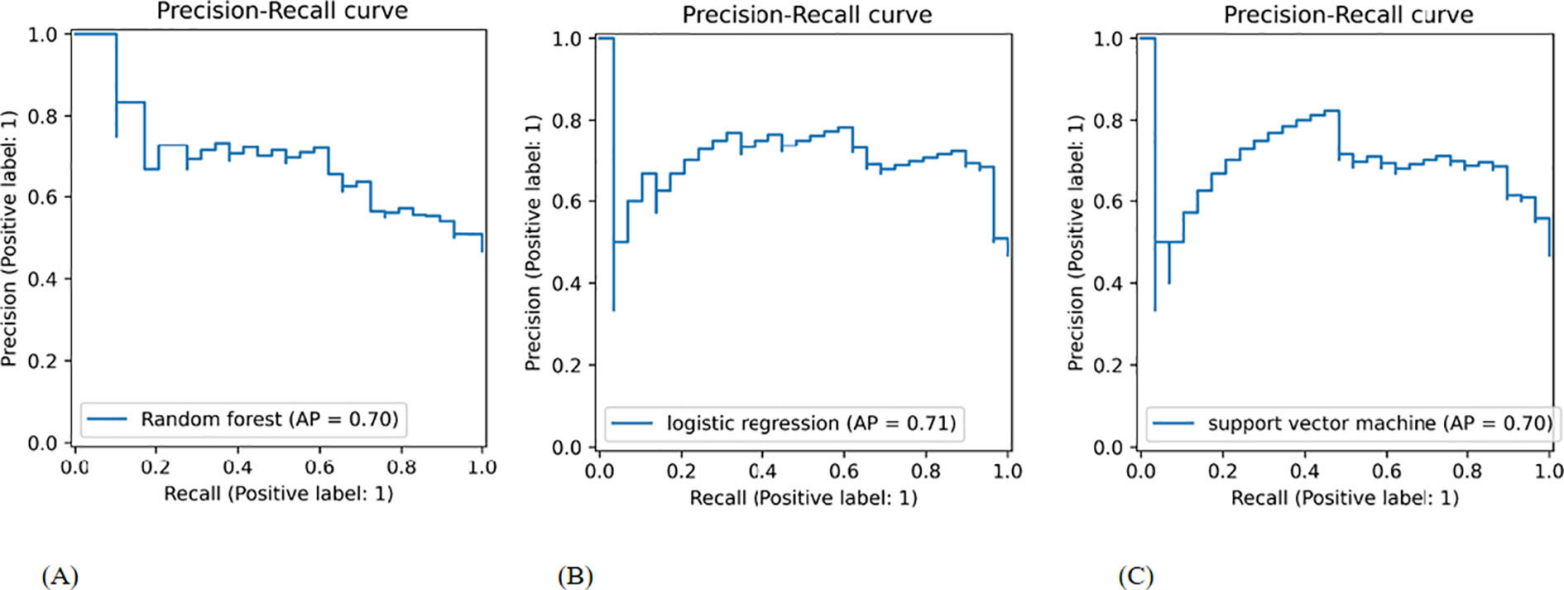
Precision–recall curves of the Cli-DLR models in the validation cohort: **(A)** random forest; **(B)** logistic regression; **(C)** support vector machine.

**Figure 6:**
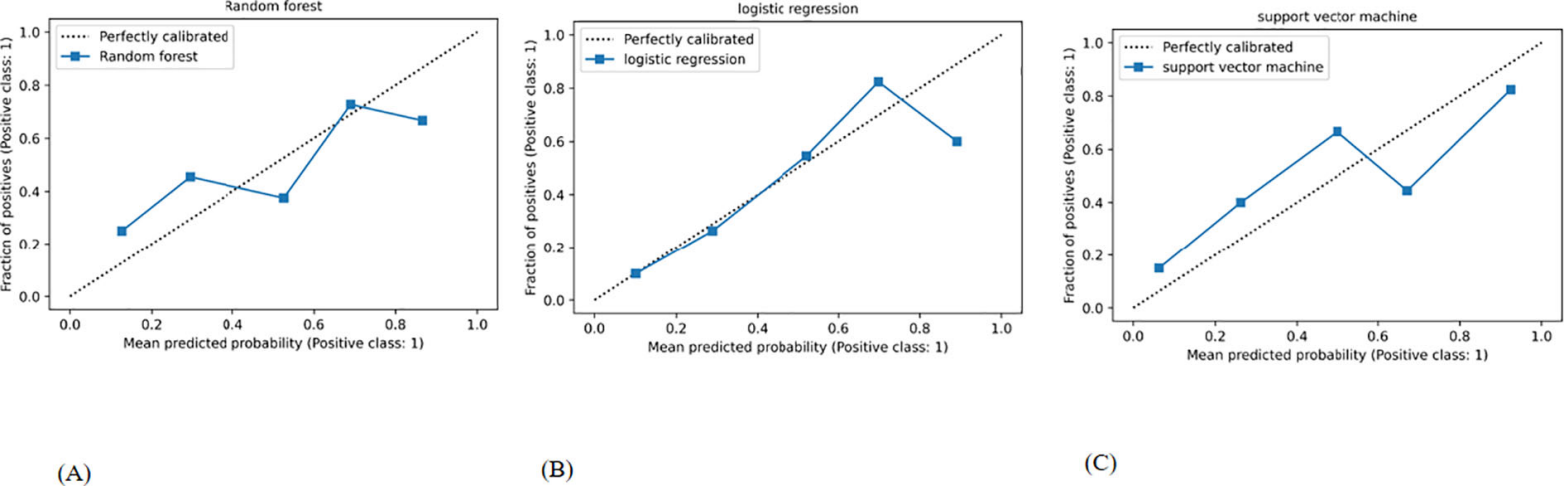
Calibration curves of the Cli-DLR models in the validation cohort: **(A)** random forest; **(B)** logistic regression; **(C)** support vector machine.

**Figures 5A-C** and **6A-C** illustrates the use of precision-recall curves and calibration curves to assess the performance of the Cli-DLR models.

In medical situations, missing a positive case (a false negative) can have serious consequences, as it means that a PTC patient with CLNM may be misdiagnosed and left untreated. A high recall shows that the model is effectively capturing the majority of the positive cases (38, 39). Precision plays a crucial role in medical diagnosis by reflecting the accuracy of identifying positive cases. Specifically, in predicting the presence of CLNM in PTC, a high precision signifies that when the model identifies someone as having CLNM, it is highly likely to be accurate.

This emphasis on precision is vital to avoid unnecessary treatments or interventions for individuals who do not possess the condition (39, 40). In the stairstep segment depicted in **Figure 5**, there is an inverse correlation between the recall and precision. At the edges of these steps, even a slight tweak in the threshold can notably impact precision while marginally enhancing recall. By analyzing the precision–recall relationship of the Cli-DLR model (**Figure 5B**), we observe an average precision of 0.71, indicating satisfactory performance. While both metrics hold significant importance, it is imperative to achieve a harmonious balance between them. The F1 score, a composite metric integrating precision and recall, serves as a valuable tool for evaluating a model’s overall performance, particularly in scenarios with imbalanced class distributions. In this study, the F1 score of the Cli-DLR was determined to be 0.68 within the validation cohort, representing a satisfactory level of performance.

The calibration curve of a prediction model serves as a crucial measure for assessing the accuracy of disease risk prediction in predicting individual outcomes. A strong calibration indicates precise predictions, while a weaker calibration suggests potential overestimation or underestimation of illness risk. The blue line illustrates the performance of the machine learning algorithms, while the diagonal dotted line serves as an ideal prediction reference (**Figure 6**). A closer alignment with the diagonal dotted line indicates a more accurate prediction. Calibration curves in proximity to the diagonal line, as seen with the Cli-DLR model (**Figure 6B**), signify strong concordance between the actual CLNM status and the predicted probability.

**Figure 7:**
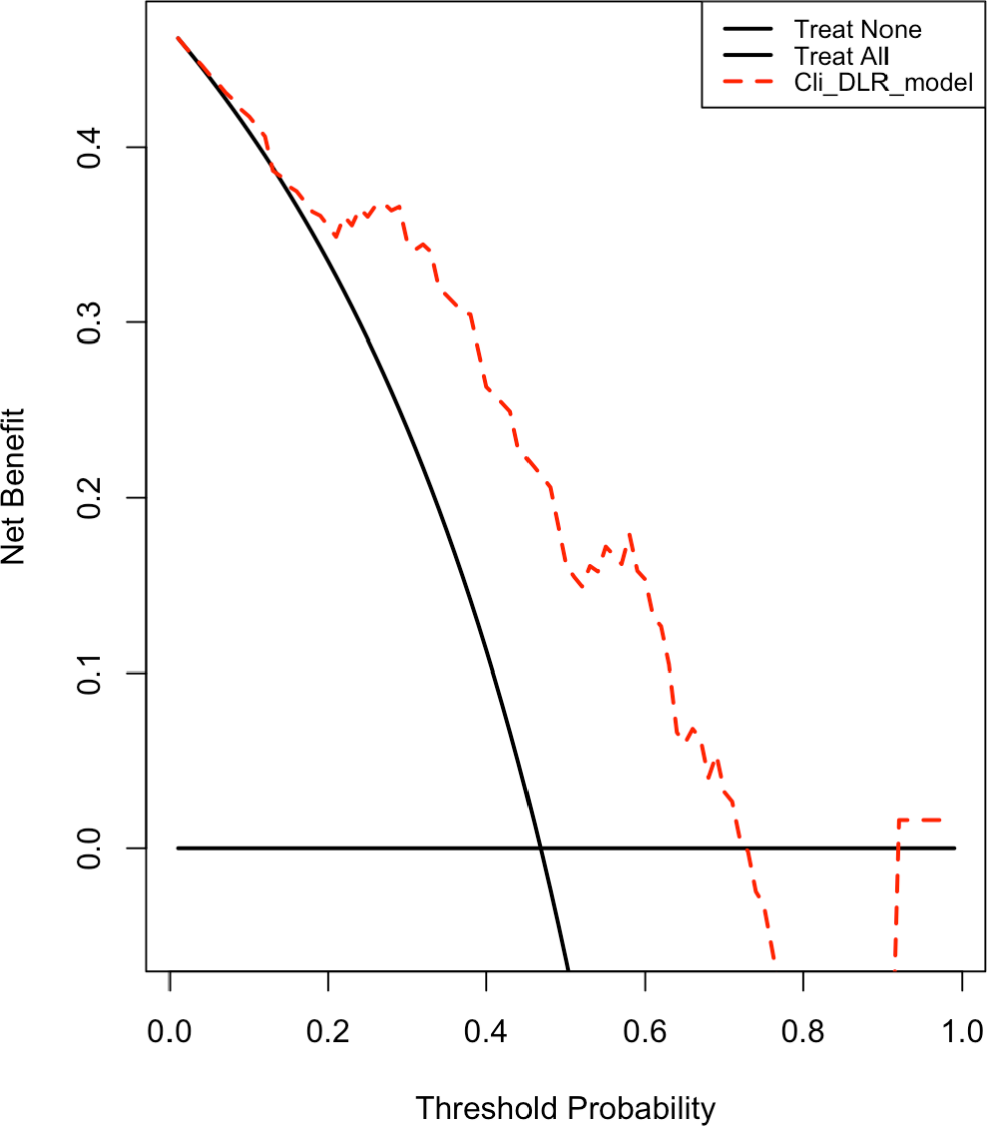
Decision curve analysis of the Cli-DLR model.

Decision curve analysis (**Figure 7**) revealed that the Cli_DLR_model yielded a greater net benefit than both the treat-all and treat-none strategies across a clinically pertinent range of threshold probabilities for predicting CLNM in patients with PTC. The model demonstrated a consistent positive net benefit ranging from approximately 0.05 to 0.65, signifying enhanced clinical decision-making within this range. The treat-all strategy demonstrated a gradual decrease in net benefit as the threshold probability increased, becoming disadvantageous beyond approximately 0.45. At elevated threshold probabilities (>0.7), the net benefit of the Cli_DLR_model neared zero and turned negative, indicating restricted clinical utility in high-threshold contexts. The findings suggest that the Cli_DLR_model may provide significant advantages in directing risk-adapted management of CLNM in PTC patients.

## Discussion

For clinical treatment to be effective, accurate preoperative CLNM prediction in PTC patients is critical, particularly for surgeons to determine the extent of surgical resection and the requirement for CLND (41–43).

DLR has grown in popularity in recent years, and it is extremely replicable. This is because it is less influenced by interobserver differences. Currently, some studies on LNM in breast cancer patients have been performed using DLR. For instance, Liu et al. (18) developed and validated a DLR nomogram for the preoperative evaluation of axillary lymph node metastasis status in patients with newly diagnosed unifocal breast cancer. Zheng et al. (19) also reported the DLR of conventional US and shear wave elastography of breast cancer for predicting axillary lymph node metastasis status preoperatively in patients with early-stage breast cancer. However, there have been few studies on the use of the DLR to evaluate CLNM in PTC patients.

Three main models were developed and validated in this study: a DLR model based only on DLR features extracted from US images, a clinical model based only on clinical and US findings, and a combined model (Cli-DLR) model, which was the primary focus of this study for the preoperative prediction of CLNM status in patients with PTC. The CLi-DLR model, represented by RFE+LR (AUC: 0.80), and the DLR model (RFE+SVM; AUC: 0.78) outperformed the clinical model (RFE+RF; AUC: 0.66) in distinguishing PTC patients without CLNM from those with CLNM (p=0.00). That is, when selected DLR features were combined with the selected clinical parameters, CLNM prediction in our validation cohort improved significantly compared to that in the clinical model that did not include DLR features.

Moreover, the AUC of the Cli-DLR model significantly surpassed that of a seasoned radiologist with over 15 years of experience in thyroid ultrasound imaging. This radiologist’s US-reported assessment of CLNM for detecting suspicious malignant cervical lymph nodes had an AUC of 0.568 (**Supplementary Figure 4**), a sensitivity of 0.500, a specificity of 0.636, an NPV of 0.581, and a PPV of 0.557.

An AUC of 0.80 is generally regarded as indicative of good diagnostic accuracy, implying that the model can effectively stratify patients based on their metastatic risk. Nonetheless, discrimination by itself does not inherently result in clinical utility. Consequently, decision curve analysis was conducted to assess the net clinical benefit of the model across various threshold probabilities.

The decision curve analysis revealed that the Cli_DLR_model yielded a superior net benefit compared to both treat-all and treat-none strategies across clinically significant thresholds, suggesting that its predictions can substantially enhance clinical decision-making. This indicates that the model is both statistically reliable and practically beneficial for informing management decisions, such as pinpointing patients who could gain from prophylactic CLN dissection while circumventing unnecessary surgery in low-risk individuals. The model’s capacity to balance sensitivity and specificity is clinically significant, considering the potential complications of overtreatment, such as recurrent laryngeal nerve injury and hypoparathyroidism.

The Cli DLR model has shown clinical utility and good discrimination (AUC = 0.80), suggesting it could be useful as a decision-support tool for PTC patients’ individualized CLNM management. The generalizability and robustness of the results should be confirmed through additional validation in separate cohorts. From a medical standpoint, determining the extent of surgery for patients with PTC requires precise preoperative prediction of CLNM. The potential for problems like hypoparathyroidism and recurrent injury to the laryngeal nerve makes prophylactic CLN node dissection a contentious topic. Clinicians may find the suggested model useful for reducing overtreatment in low-risk patients and identifying those at increased risk of metastasis who may benefit from more aggressive surgical management. When combined with clinical variables, deep learning radiomics offers a more thorough evaluation in this setting than either traditional imaging or clinical evaluation on their own.

Yao et al. (6) created and validated a multimodal deep learning model called DeepThy-Net to predict various CLNM patterns in PTC patients. Their experimental results suggested that the AUC was between 0.870 and 0.905, demonstrating therapeutic relevance. In comparison to the current study, our Cli-DLR had a lower AUC of 0.80. The somewhat higher AUCs in the previous study could be ascribed to the increased number of patients involved in the study. Machine learning and deep learning models perform better with more datasets.

In a previous study (41), we created a clinical-ultrasound radiomic model that incorporates clinical risk variables as well as traditional handcrafted radiomic features for predicting CLNM in PTC patients, with an AUC of 0.71. Compared to that in the current study, the AUC of the previous model for CLNM diagnosis was lower (0.71 vs. 0.80). This could be attributed to the DLR method used in the current work. The drawbacks of manually crafted features are rooted in the need for human labelling and their inability to adapt precisely to a particular task (16). In contrast, DLR (15) is a novel technique that can autonomously unveil several levels of representations adapted to specific prediction challenges using end-to-end learning. Moreover, deep learning algorithms learn high-level features from data incrementally, which is their main advantage. This decreases the demand for domain expertise and the extraction of hardcore features.

In their study, Tian et al. (44) created a CLNM prediction algorithm based on clinical risk factors. The AUC of their model for diagnosing CLNM in the validation cohort was 0.70. Our clinical model showed an AUC of 0.66, which is consistent with that of a previous study, demonstrating that clinical risk variables alone cannot adequately assess CLNM in PTC patients. Precision-recall is a metric for measuring the quality of a model’s output. Recall is important in the context of CLNM prediction since it measures the model’s capacity to properly identify all occurrences of real CLNM situations.

Our study has several limitations that warrant attention. First, it was conducted at a single center with a relatively small dataset, which may limit the accuracy and generalizability of the developed model. To address this, we applied data augmentation, feature selection, and transfer learning techniques. Second, the ultrasound images were acquired using equipment from different manufacturers. Variations in image texture, contrast, and noise characteristics across systems may influence the extraction and stability of deep learning radiomics (DLR) features. While including multi-vendor data can improve real-world applicability, such heterogeneity may also affect feature reproducibility and model performance. In future studies, we plan to implement standardized imaging protocols, conduct external validation using datasets from diverse ultrasound platforms, and incorporate semi-automatic segmentation to further assess the robustness and generalizability of our model.

## Conclusion

In conclusion, we developed a Cli-DLR model to assess the cervical lymph node status of PTC patients prior to surgery. Radiologists and the clinical models were outperformed by the Cli-DLR model. Consequently, this model can provide a possible noninvasive method for detecting CLNM and aid in clinical decision-making due to its favorable specificity and sensitivity. High-level evidence for clinical use in later studies is anticipated to be obtained through prospective multicenter validation.

## Data Availability

The original contributions presented in the study are included in the article/**Supplementary Material**. further inquiries can be directed to the corresponding author.
