# Supplementary material for: Ultrasound deep learning features for predicting cervical lymph node metastasis in papillary thyroid carcinoma
Source: Front Oncol. 2026 May 20;16:1721234. doi: 10.3389/fonc.2026.1721234 (PMC13229823; doi:10.3389/fonc.2026.1721234)

**Supplementary Method Evaluation metrics employed in this study**

The models' performance on both the training and validation datasets was evaluated using metrics such as the area under the curve (AUC), confusion matrix, precision, sensitivity, specificity, negative predictive value (NPV), positive predictive value (PPV), accuracy, precision‒recall curves, F1 score, and other standard clinical statistics. The best-performing model was selected from a pool of models based on its performance on the validation dataset, which was evaluated and compared for diagnostic efficacy. In CLNM prediction, the confusion matrix provides a concise overview of the model's performance, juxtaposing its predictions against the real outcomes.

The ROC curve plots the true positive rate (sensitivity) against the false positive rate (1 – sensitivity), and the AUC can be calculated. The accuracy, in the context of CLNM prediction, gauges the model's overall accuracy in predicting the presence or absence of CLNM in individuals with PTC. The accuracy can be calculated from Eqn (7)[1]. Precision in the context of CLNM prediction refers to the ratio of accurately predicted CLNM cases to all cases that the model presented as having CLNM. Eqn. (5) determines the precision [1]. The F1 score (Eqn. 6) balances precision and recall in the context of CLNM prediction by offering a harmonic mean that encompasses both false positives and false negatives [2]. Recall or sensitivity (Eqn. 4 and 7) for CLNM prediction represents the ratio of correctly predicted cases of CLNM out of all actual cases of CLNM based on the true positive rate, showing a model's capacity to correctly identify all people who have CLNM [2].

In the context of CLNM prediction, specificity (Eqn. 9) is described in a variety of ways, including a model's capacity to detect true negatives, being based on the true negative rate, and properly identifying those who do not have CLNM. These evaluation metrics play a crucial role in assessing the efficiency and effectiveness of machine learning models. In Equations 4, 5, 7, 8, 9, 10 and 11, *TP* represents the number of true positive predictions (correctly predicted positive CLNM), *FP* represents the number of false positive predictions (incorrectly predicted positive CLNM), *TN* represents the number of true negative predictions (correctly predicted negative CLNM), and *FN* represents the number of false negative predictions (incorrectly predicted negative CLNM). These metrics provide valuable insights into the accuracy and performance of CLNM predictions.

(4)

(5)

(6)

(7)

(8)

(9)

(10)

(11)

**Supplementary References**

[1] Md. A. Islam, Md. Z. H. Majumder, and Md. A. Hussein, “Chronic kidney disease prediction based on machine learning algorithms,” *J. Pathol. Inform.*, vol. 14, p. 100189, Jan. 2023, doi: 10.1016/j.jpi.2023.100189.

[2] Md. Ziaul Hasan Majumder, Md. Abu Khaer, Md. J. Nayeen Mahi, Md. Shaiful Islam Babu, and S. K. Aditya, “Decision Support Technique for Prediction of Acute Lymphoblastic Leukemia Subtypes Based on Artificial Neural Network and Adaptive Neuro-Fuzzy Inference System,” in *Inventive Systems and Control*, vol. 204, V. Suma, J. I.-Z. Chen, Z. Baig, and H. Wang, Eds., in Lecture Notes in Networks and Systems, vol. 204. , Singapore: Springer Singapore, 2021, pp. 539–554. doi: 10.1007/978-981-16-1395-1_40.

**Supplementary Table 1 Clinical variables selected after PCC and RFE analysis**

| Features  age  mass  Tumour internal vascularization  Capsular invasion  Aspect ratio  Shape  Internal echo  Ultrasound ETE diagnosis  ultrasound CLNM diagnosis | Coefficient  -0.853672497  1.25578738  0.878927061  -0.345555868  -0.477549311  0.49622969  0.643579647  0.227435645  0.695144309 |
| --- | --- |

**Supplementary Table 2 Deep learning radiomic features selected after PCC and RFE analysis**

| Features  f_38  f_162  f_203  f_234  f_272  f_354  f_470  f_526  f_561  f_763  f_789  f_887  f_913  f_1235  f_1332  f_1349  f_1407  f_1422  f_1534  f_1587  f_1630  f_1651  f_1683  f_1706  f_1753  f_1840  f_1883  f_1940  f_1972  f_2034 | Coefficient  -0.452574454  -0.663490191  -0.796323818  -0.630012924  1.036246752  0.552170671  -0.775585493  -0.893458893  0.787617189  -0.580318692  1.15758261  0.787883818  0.675083659  -0.707600971  -0.614567978  1.2989278  0.885193442  -0.887634211  -0.677417327  -0.738273933  -0.732803855  -0.742645836  -0.640798347  -0.755981821  -0.720364473  -0.821587792  0.520975513  -0.633195722  -0.918861093  0.72160685 |
| --- | --- |

**Supplementary Table 3 selected features after PCC and RFE for constructing the combined models**

| Features  f_38  f_162  f_203  f_234  f_272  f_354  f_470  f_526  f_561  f_763  f_789  f_887  f_913  f_1235  f_1332  f_1349  f_1407  f_1422  f_1534  f_1587  f_1630  f_1651  f_1683  f_1706  f_1753  f_1840  f_1883  f_1940  f_1972  f_2034  age  mass  Tumour internal vascularization  Capsular invasion  Aspect ratio  Shape  Internal echo  Ultrasound ETE diagnosis  ultrasound CLNM diagnosis | Coefficient  -0.452574454  -0.663490191  -0.796323818  -0.630012924  1.036246752  0.552170671  -0.775585493  -0.893458893  0.787617189  -0.580318692  1.15758261  0.787883818  0.675083659  -0.707600971  -0.614567978  1.2989278  0.885193442  -0.887634211  -0.677417327  -0.738273933  -0.732803855  -0.742645836  -0.640798347  -0.755981821  -0.720364473  -0.821587792  0.520975513  -0.633195722  -0.918861093  0.72160685  -0.853672497  1.25578738  0.878927061  -0.345555868  -0.477549311  0.49622969  0.643579647  0.227435645  0.695144309 |
| --- | --- |

**Supplementary Figure 1** Recursive feature elimination (RFE) with 15‐fold cross‐validation; number of features selected vs. cross‐validation score. (A) Clinical model. (B) DLR model.

**
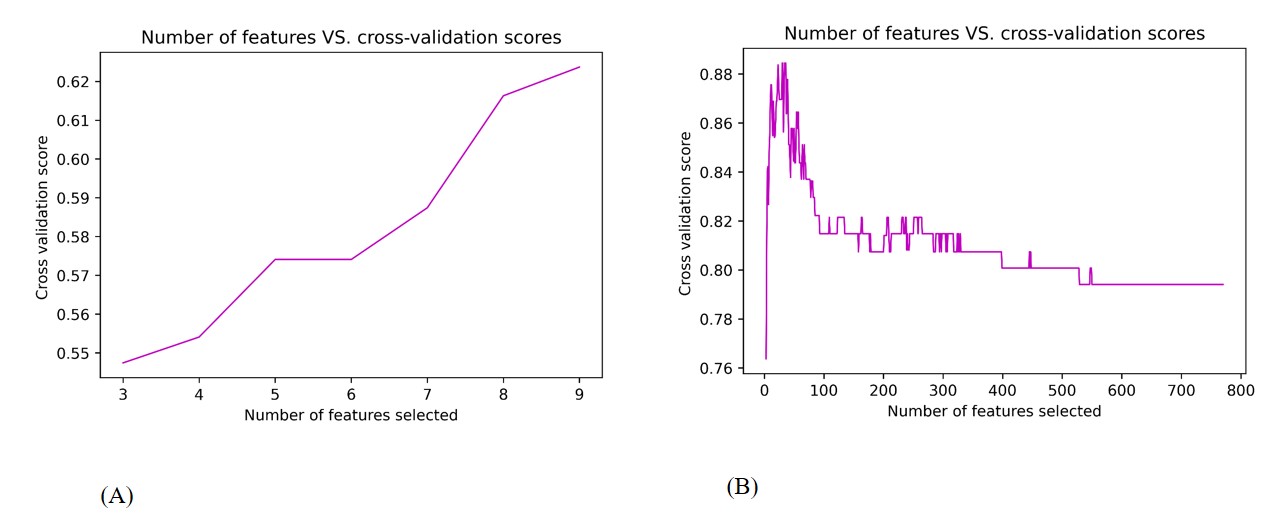
**

**Supplementary Figure 2** ROC curves of the classifiers used in building the clinical model employing RFE. ROC, receiver operating characteristic. (A) Random forest (B) Logistic regression classifier. (C) Support vector machine.


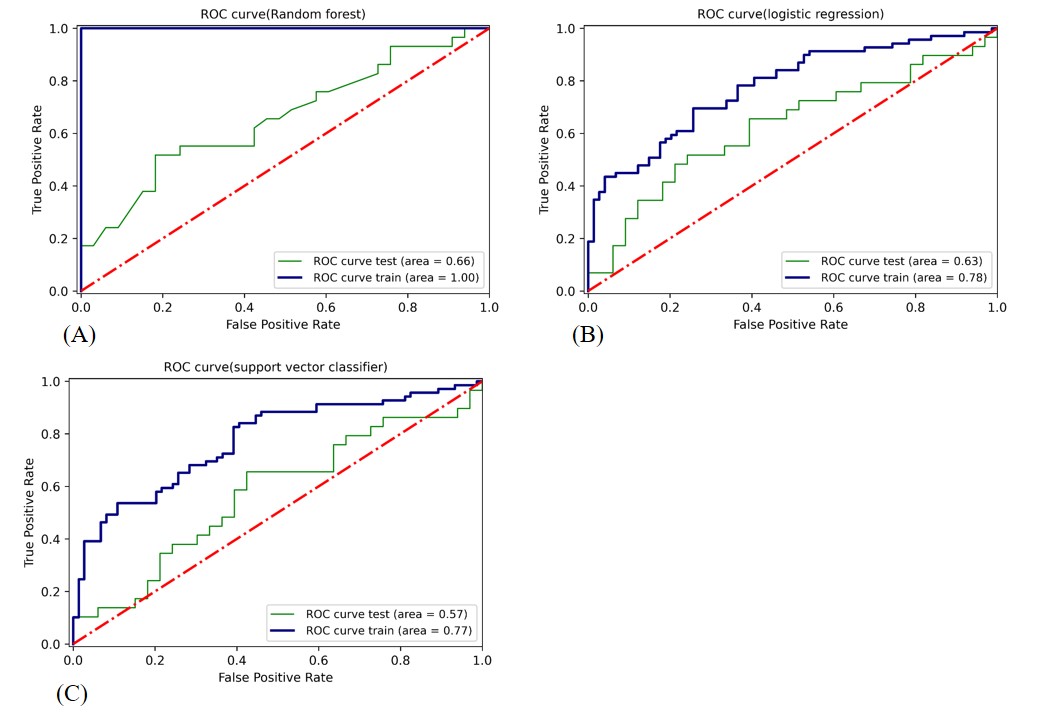


**Supplementary Figure 3** ROC curves of the classifiers used in building the DLR model employing RFE. ROC, receiver operating characteristic. (A) Random forest; (B) logistic regression classifier; (C) support vector machine.


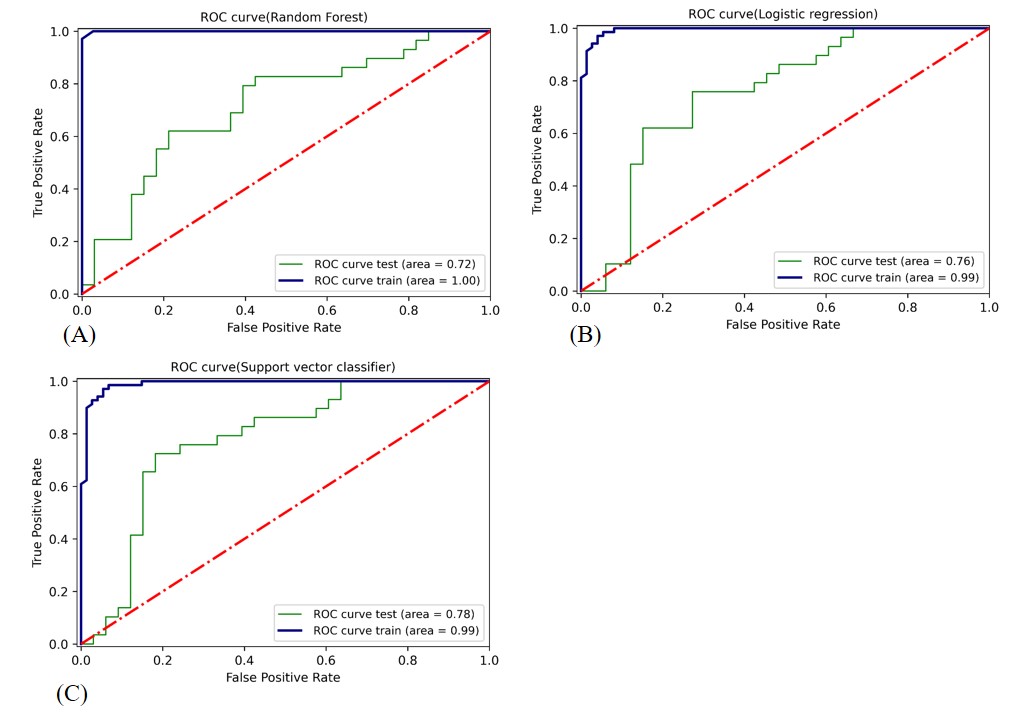


**Supplementary Figure 4** ROC curve of US-reported CLNM status by an experienced sonographer. ROC, receiver operating characteristic.


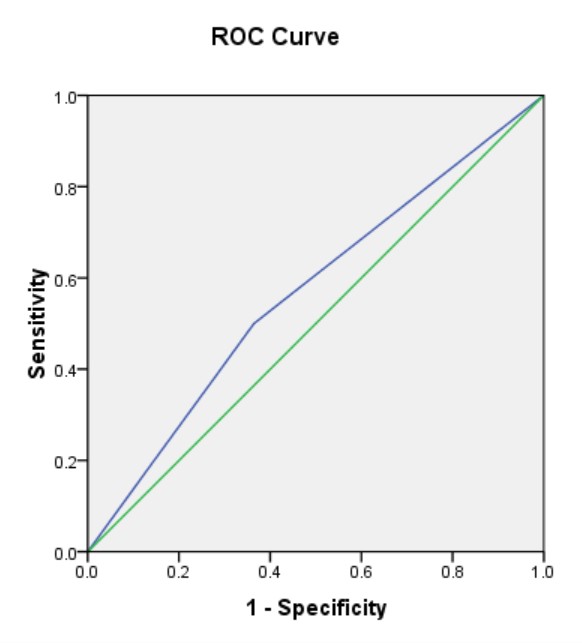

Supplement: Supplementary file 1 [file DataSheet1.doc]
